# Increased Expression of 11β-Hydroxysteroid Dehydrogenase Type 1 Contributes to Epidermal Permeability Barrier Dysfunction in Aged Skin

**DOI:** 10.3390/ijms22115750

**Published:** 2021-05-27

**Authors:** Beom Jun Kim, Noo Ri Lee, Chung Hyeok Lee, Young Bin Lee, Sung Jay Choe, Solam Lee, Hyun Jee Hwang, Eunjung Kim, Gareth G. Lavery, Kyong-Oh Shin, Kyungho Park, Eung Ho Choi

**Affiliations:** 1 Department of Dermatology, Yonsei University Wonju College of Medicine, Wonju 26426, Korea; kimbeomjun11@naver.com (B.J.K.); noorie00@naver.com (N.R.L.); nitr_o@naver.com (C.H.L.); lyb032@naver.com (Y.B.L.); wow8561@naver.com (S.J.C.); esolami@naver.com (S.L.); 01181129@hanmail.net (H.J.H.); hyksuj0326@naver.com (E.K.); 2 Institute of Metabolism and Systems Research, College of Medical and Dental Sciences, University of Birmingham, Birmingham B15 2TT, UK; g.g.lavery@bham.ac.uk; 3 Department of Food Science and Nutrition, Convergence Program of Materials Science for Medicine and Pharmaceutics, Hallym University, Chuncheon 24252, Korea; 0194768809@hanmail.net (K.-O.S.); hopark78@gmail.com (K.P.)

**Keywords:** barrier function, skin aging, 11-beta-hydroxysteroid dehydrogenase type 1, glucocorticoids, epidermal lipids

## Abstract

Inactive cortisone is converted into active cortisol by 11β-hydroxysteroid dehydrogenase type 1 (11β-HSD1). Excessive levels of active glucocorticoids could deteriorate skin barrier function; barrier impairment is also observed in aged skin. In this study, we aimed to determine whether permeability barrier impairment in the aged skin could be related to increased 11β-HSD1 expression. Aged humans (n = 10) showed increased cortisol in the stratum corneum (SC) and oral epithelium, compared to young subjects (n = 10). 11β-HSD1 expression (as assessed via immunohistochemical staining) was higher in the aged murine skin. Aged hairless mice (56-week-old, n = 5) manifested greater transepidermal water loss, lower SC hydration, and higher levels of serum inflammatory cytokines than the young mice (8-week-old, n = 5). Aged 11β-HSD1 knockout mice (n = 11), 11β-HSD1 inhibitor (INHI)-treated aged wild type (WT) mice (n = 5) and young WT mice (n = 10) exhibited reduced SC corticosterone level. Corneodesmosome density was low in WT aged mice (n = 5), but high in aged 11β-HSD1 knockout and aged INHI-treated WT mice. Aged mice exhibited lower SC lipid levels; this effect was reversed by INHI treatment. Therefore, upregulation of 11β-HSD1 in the aged skin increases the active-glucocorticoid levels; this suppresses SC lipid biosynthesis, leading to impaired epidermal permeability barrier.

## 1. Introduction

Various physiological parameters in aged skin, including structure, wound healing ability, immune function, and metabolism, show changes compared to those in young skin [1,2,3,4,5]. In addition, the skin barrier function reportedly deteriorates with an increased surface pH, leading to impaired skin integrity and cohesion and delayed barrier recovery, due to the reduction of epidermal lipid synthesis [6,7,8]. These characteristics of the aged skin are similar to the changes caused by excessive endogenous or exogenous glucocorticoid (GC) levels [2]. Apart from the topical GC administration-induced increase in the local GC concentration, systemic GC accumulation (e.g., in Cushing syndrome or psychological stress) could also trigger such changes [9,10,11]. Moreover, different types of stresses (not only psychologic, but also physiologic and physical stress) and stress-associated increase in GC levels reportedly play an important role in skin homeostasis and various skin diseases [12,13].

Excessive GC levels suppress skin barrier function through mechanisms such as the inhibition of keratinocyte proliferation and differentiation or the suppression of epidermal lipid synthesis [9]. Therefore, physiological changes in the aged skin might be associated with the increase of the active GC level [2,14,15,16].

Cortisol, an active GC in the human blood, is mainly generated in the adrenal cortex upon the stimulation of the hypothalamic–pituitary–adrenal (HPA) axis. The skin is also known to act as an endocrine organ equivalent to the HPA axis [17,18,19,20,21,22]. GCs could be synthesized from cholesterol in the skin [23,24]. The de novo local cortisol production pathway involves 11β-hydroxysteroid dehydrogenase type 1 (11β-HSD1); this enzyme converts inactive cortisone to active cortisol, which is expressed in several tissues including the skin, liver, kidneys, large intestine, bone, skeletal muscle, and adipose tissue [25,26,27,28]. The local GC level is predominantly regulated via the activity of 11β-HSD1 [29]. This enzyme is believed to participate in skin homeostasis through the regulation of the endogenous GC levels [30,31,32] and has been implicated in delayed wound healing and the inhibition of keratinocyte proliferation and differentiation.

The skin is always exposed to physical or chemical irritants; these irritants affect aged skin to a larger extent due to its reduced barrier function compared to the young skin. Local GC activation may be important for controlling local stressors in the irritation-susceptible aged skin. The 11β-HSD1 level is known to increase with aging and wound healing [15,33], and this phenomenon may represent a mechanism underlying GC activation in response to aging-induced epidermal stress. This mechanism is a possible explanation for the decreased prevalence of atopic dermatitis (a representative inflammatory skin disease) [34] and the increased prevalence of contact dermatitis and xerotic eczema in the elderly [35,36]. Recently, we have reported that 11β-HSD1 is a major factor that affects the pathophysiology of atopic dermatitis via the suppression of atopic inflammation through the modulation of active GC levels in the skin [37]. Several studies report on the effects of 11β-HSD1 on GC upregulation and skin aging. The suppression of 11β-HSD1 expression reduces the cutaneous adverse effects of excessive GC levels and reverses the aging-induced alterations of skin functions [2,38,39,40].

Therefore, we hypothesized that increased 11β-HSD1 expression and the subsequent increase in the GC levels contribute to the deterioration of barrier function in the aged skin. We tested this hypothesis by analyzing the influence of 11β-HSD1-mediated regulation of the endogenous GC levels on the barrier function of the aged skin.

## 2. Results

### 2.1. Stratum Corneum (SC) and Oral Epithelium Cortisol Levels Are Higher in the Aged Participant

**Figure 1:**
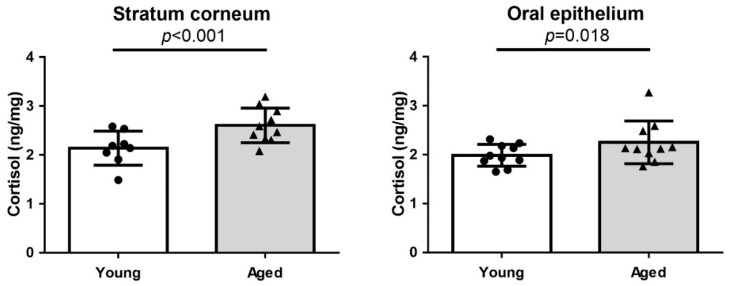
Cortisol levels in the stratum corneum (SC) (**left**) and oral epithelium (**right**) collected from young and aged participants (n = 10 each). The cortisol levels both in the SC of the forearms and in the oral epithelium of the buccal mucosa were significantly higher in the aged group than in the young group. The data are presented as the means ± SD.

The age of the participants in the young and old groups were 21.6 ± 1.89 and 65.9 ± 4.10 years (mean ± SD, *p* < 0.001), respectively. The cortisol level in the SC collected from the forearms was significantly higher in the aged group than in the young group (2.604 ± 0.3438 vs. 2.139 ± 0.3359 ng/mg, *p* < 0.001). In addition, significantly higher cortisol levels were detected in the buccal mucosa epithelium of the aged group than in that of the young group (2.252 ± 0.4250 vs. 1.988 ± 0.2183 ng/mg, *p* = 0.018; Figure 1).

### 2.2. Increased 11β-HSD1 Expression in the Skin of Aged Hairless Mice

**Figure 2:**
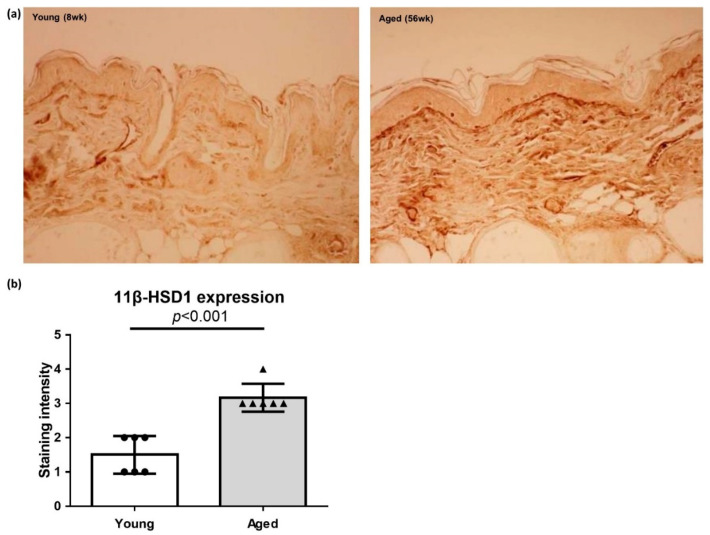
Immunohistochemical (IHC) staining and semi-quantitative analysis of the 11β-hydroxysteroid dehydrogenase type 1 (11β-HSD1) expression in the skin of young and aged hairless mice. (**a**) The 11β-HSD1 expression is higher in the skin of aged hairless mice than in that of young mice. (**b**) The results of the semi-quantitative analysis, showing significantly higher 11β-HSD1 expression in the aged skin than in the young skin (n = 6 each). The data are presented as the means ± SD.

The immunohistochemical (IHC) staining revealed higher 11β-HSD1 expression in the skin of aged hairless mice than in that of young mice (Figure 2a). In particular, high 11β-HSD1 expression was observed in the basal layer of the epidermis and dermis as well as in the SC. The semiquantitative analysis also showed significantly higher 11β-HSD1 expression in the aged skin (3.167 ± 0.4082 vs. 1.5 ± 0.5477, *p* < 0.001; Figure 2b).

### 2.3. Deterioration of Skin Barrier Function in Aged Hairless Mice

**Figure 3:**
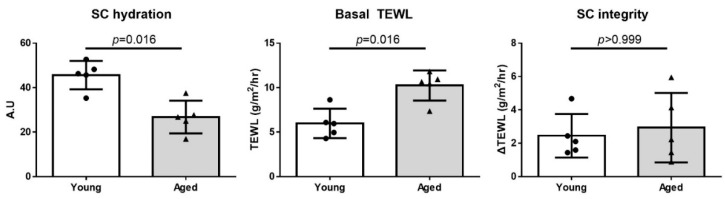
Skin barrier function in the skin of young and aged hairless mice. The aged mice had lower SC hydration and higher basal TEWL, but there was no difference in SC integrity between the groups. SC integrity was defined as the change in TEWL after 15 rounds of D-Squame disc tape stripping. The data are presented as the means ± SD. SC, stratum corneum; TEWL, transepidermal water loss.

We evaluated the skin barrier function of young (8-week-old) and aged (56-week-old) hairless mice (Figure 3). The aged mice featured significantly higher transepidermal water loss (TEWL; 9.630 ± 2.1273 vs. 5.980 ± 1.6571 g/[m^2^⋅h], *p* = 0.016) and lower SC hydration (27.09 ± 6.626 vs. 45.64 ± 6.409 arbitrary units (AU), *p* = 0.016) than the young mice. Nonetheless, we detected no difference in the SC integrity between the groups (2.448 ± 1.3049 vs. 3.195 ± 1.9749 g/[m^2^⋅h], *p* = 0.999).

### 2.4. Increased Serum Inflammatory Cytokine Levels in Aged Hairless Mice

**Figure 4:**
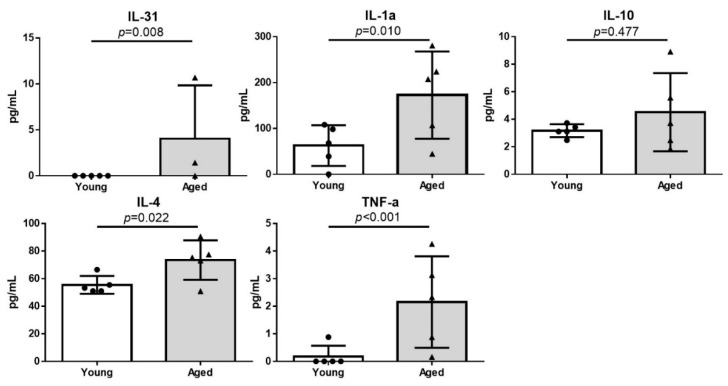
Inflammatory cytokine levels in the serum of young and aged hairless mice. Concentrations of inflammatory cytokines were significantly higher in the serum of aged mice. The data are presented as the means ± SD. IL, interleukin; TNF, tumor necrosis factor.

We measured the serum cytokine levels in the young and aged hairless mice using enzyme-linked immunosorbent assays (ELISAs). The levels of inflammatory cytokines, including interleukin (IL) 31, IL-1α, IL-4, and tumor necrosis factor-α (TNF-α) were significantly higher in the serum of aged mice (Figure 4).

### 2.5. Reduced SC Corticosterone Concentration in Aged 11β-HSD1 (HSD11B1) Knockout (KO) Mice and Aged 11β-HSD1 Inhibitor (INHI)-Treated Wild-Type (WT) Mice

**Figure 5:**
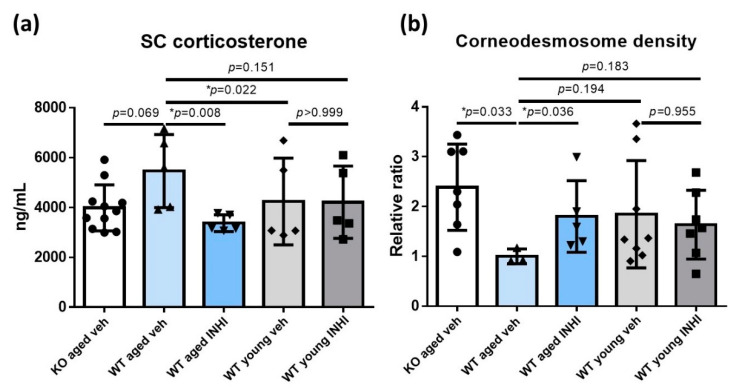
Stratum corneum (SC) corticosterone and corneodesmosome (CD) density in HSD11B1 knock-out (KO) and wild type (WT) mice. (**a**) The SC corticosterone level was the highest in the aged WT mice. Both the HSD11B1 KO and treatment with the 11β-HSD1 inhibitor (INHI) decreased the SC corticosterone level in the aged mice. (**b**) The CD density was the highest in the aged KO mice. The aged WT mice showed lower CD density than the young WT mice. INHI treatment increased the CD density in the aged WT mice. The data are presented as the means ± SD. * statistically significant (*p* < 0.05); veh, vehicle.

The SC corticosterone levels were the highest in the aged WT mice (5461.94 ± 1465.21 ng/mL). Compared to these, the SC corticosterone levels were lower in the young WT mice (4244.20 ± 1741.36 ng/mL, *p* = 0.022) and the aged HSD11B1 KO mice (3987.51 ± 922.88 ng/mL, *p* = 0.069). The SC corticosterone levels were also low in the aged WT mice treated topically with INHI (3373.46 ± 333.61, *p* = 0.008). The reduction in the SC corticosterone level by topical INHI treatment was rather modest in young WT mice (Figure 5a and Table S1).

### 2.6. Decreased Corneodesmosome (CD) Density in WT Aged Mice Compared to That in Aged HSD11B1 KO Mice and Aged INHI-Treated WT Mice

The CD density was measured by electron microscopic (EM) imaging to evaluate the SC integrity (Figure 5b and Table S2). The calculation of the CD density ratio in aged WT mice showed a significantly higher CD density in the aged KO mice (the ratio of 2.39, *p* = 0.033). The CD density in the young WT mice was higher than that in the aged WT mice (ratio = 1.85, *p* = 0.194); the topical INHI application led to the recovery of the CD density in the aged WT mice (ratio = 1.80, *p* = 0.036). The topical INHI application did not affect the young WT mice.

### 2.7. Recovery of the SC Lipid Levels in WT Aged Mice upon the Topical INHI Treatment

**Figure 6:**
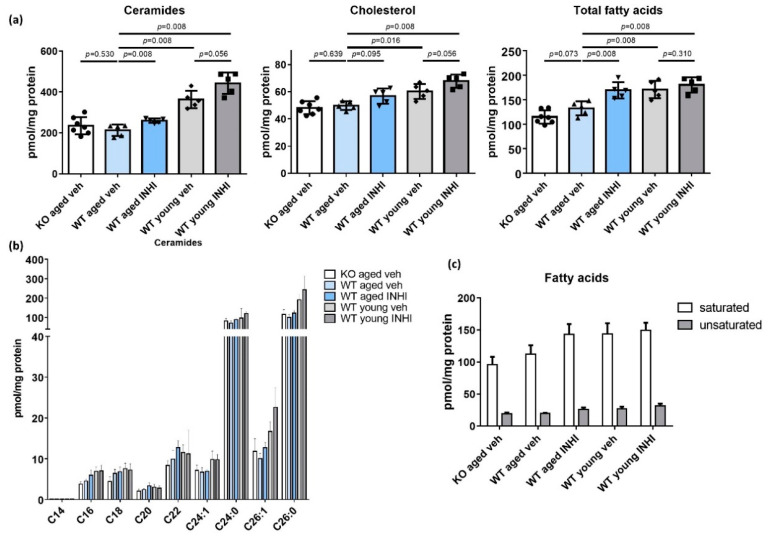
Lipid analysis of HSD11B1 knock-out (KO) and wild type (WT) mice. (**a**) The levels of three major types of SC lipids (ceramide, cholesterol, and free fatty acids (free FAs)) were significantly lower in aged WT mice than in young WT mice after treatment with the vehicle (*p* < 0.05 in each case). Topical INHI treatment led to a recovery of the levels of all three lipid types in both the aged and young WT mice. The levels of ceramide and total FAs increased significantly in the aged mice after INHI treatment (each *p* = 0.008), whereas the cholesterol level did not (*p* = 0.095). The extent of these increases was smaller among young mice. There was no difference between the levels of all three lipid types in the aged HSD11B1 KO mice and aged WT mice. (**b**) The ceramide levels were evenly reduced in the aged WT and aged HSD11B1 KO mice regardless of the number of carbons in the fatty acyl chains. (**c**) In the aged mice, free FA levels were also low regardless of the presence of saturation. The data are presented as the means ± SD. INHI, 11β-HSD1 inhibitor; veh, vehicle.

The levels of all three major types of SC lipids (ceramide, cholesterol, and free fatty acids (free FAs)) were significantly lower in the aged WT mice than in the young WT mice after treatment with the vehicle (*p* < 0.05 in each case; Figure 6a and Tables S3–S5). Topical treatment with INHI led to the recovery of the levels of all three lipid types in both aged and young WT mice. In particular, the aged mice showed significantly higher ceramide and total FA levels upon INHI treatment (*p* = 0.008 in each case), though the changes in cholesterol levels were not significant (*p* = 0.095). The extent of this increase was less drastic in young mice. There was also no difference between the levels of all three lipid types in the aged HSD11B1 KO and aged WT mice. Ceramide levels were similarly lower in both the WT and KO aged mice regardless of the number of chains (Figure 6b). The free FA levels were also lower, regardless of saturation, in aged mice (Figure 6c).

## 3. Discussion

Increased active GC levels reportedly deteriorate the skin barrier function by inhibiting keratinocyte differentiation and proliferation [41,42], lipid synthesis [9], and serine protease activation via an increase in the skin surface pH [43,44]. The adverse changes in the function and structure of the aged skin may be due to the increased local GC production, which is largely mediated by 11β-HSD1 [14,29]. The 11β-HSD1 expression reportedly increases with aging [14,15,40], and the present study provides evidence regarding the fact that the cutaneous activation of endogenous GCs is associated with skin aging, mediated by an increase in 11β-HSD1 expression.

In this study, we detected increased cortisol levels in both the SC and the oral mucosal cells in the aged participants (Figure 1). In our previous study on psychological stress-induced skin barrier damage [45], elevated SC cortisol levels positively correlated with the extent of skin barrier dysfunction and 11β-HSD1 concentration in the oral mucosa. Therefore, in the present study, cortisol levels were measured in both the SC and the oral mucosa. Nonetheless, we noted a lack of correlation between the SC cortisol and the oral epithelial cortisol levels. Moreover, the 11β-HSD1 level in the oral mucosa showed no age-dependency (data not shown). As 11β-HSD1 is expressed predominantly in the suprabasal layer of the epidermis and dermis, this epidermal expression could be detected only by a whole-epidermis skin biopsy [45]. In the mouse skin IHC staining assay reported in this study, the 11β-HSD1 expression was observed mainly in the basal layer of the epidermis and throughout the dermis, which was more evident in aged mice (Figure 2). We believe that the detected increase in the cortisol levels in the elderly and the higher 11β-HSD1 expression in the aged murine skin validate the first part of our hypothesis.

When we explored the differences in skin barrier function between young and aged hairless mice (Figure 3), we noted a significant reduction in SC hydration and an increase in the basal TEWL in aged mice, consistent with results from previously published studies [46]. In contrast, the SC integrity showed a statistically non-significant decrease. These observations could be explained by the fact that the aged mice used in this experiment were 56-week-old, i.e., they were relatively young compared to those used in another study [47].

The levels of inflammatory cytokines, such as IL-1α, IL-4, IL-31, and TNF-α, were significantly elevated in the serum of aged hairless mice, compared to those in the serum of young mice (Figure 4). This result is in good agreement with that of another previous study [48], which showed that sustained abnormal epidermal permeability increases the levels of inflammatory cytokines, which might render the elderly susceptible to chronic inflammatory diseases. A prolonged reduction in SC hydration also contributes to the aggravation of cutaneous inflammation, independently of the barrier disruption [49,50].

The observed increase in the SC corticosterone concentration in aged WT mice and its decrease in the aged *HSD11B1* KO mice and aged INHI-treated WT mice further support our hypothesis and validate our experiments (Figure 5a). The inconspicuous changes in the SC corticosterone levels upon the INHI treatment in young WT mice may be due to their good functional health.

The levels of all three major types of SC lipids, including ceramide, cholesterol, and free FAs, proved to be significantly lower in the aged mice than in the young mice. These lipids are the main components important for not only the epidermal barrier formation, but also the maintenance of barrier function [51]. Therefore, the changes in their profiles could correlate with an impaired skin barrier function [52]. The levels of both long- and short-chained ceramides, were lower (to a similar extent) in the aged mice. Furthermore, the levels of both saturated and unsaturated FA levels showed a decrease (to similar extents) in the aged mice (Figure 6). These observations are consistent with those of other studies [53,54]. The detected increase in the SC lipid concentrations in the aged INHI-treated mice, compared to that in the vehicle-treated group (along with significant ceramide and FA upregulation and a slight, though non-significant, increase in the cholesterol levels) validates our hypothesis. Our hypothesis also predicted higher SC lipid levels in the aged *HSD11B1* KO mice than in the aged WT mice, but this prediction was found to be incorrect, probably because *HSD11B1* KO mice can produce sufficient compensatory cortisol in the blood to inhibit epidermal lipid synthesis. The inconspicuous changes in the SC lipid concentrations after the treatment of young WT mice with INHI can be ascribed to the health of the latter, as mentioned earlier.

Intrinsic and extrinsic aging-related epidermal thinning reduces the skin barrier function [55], thereby rendering the skin susceptible to daily external physical or chemical irritation. Epidermal stressors can induce inflammatory cytokine production [48], affecting steroid synthesis in the skin [56,57] and activating the HPA axis [58,59,60]. Local GC activation is needed to control this inflammatory trend in the aged skin, similar to the case for the active GC and 11 β-HSD1 level increase in the wounded skin [33]. In addition, growth hormone, which inhibits 11β-HSD1 activity, is downregulated with age [61]. Eventually, local GC activation induced by the increase in the 11β-HSD1 levels in the aged skin contributes to the skin barrier impairment associated with the disrupted homeostatic responses of the aged skin.

In our previous experiments performed using mice, the male mice fought and damaged the skin of each other during the breeding process; this rendered the optimal control set-up difficult to establish. This problem was solved by using only female mice, which yielded reliable results. Therefore, this study included only female subjects. Although sex-dependent cortisol level variations may also need to be considered [62,63], we used only female subject to set up good controls and ease the comparison and result interpretation processes.

We believe that our results validate the hypothesis that increased 11β-HSD1 expres-sion and the subsequent GC level increase could contribute to the deterioration of barrier function in the aged skin. In this study, we observed that active GCs (such as cortisol or corticosterone) and 11β-HSD1 were upregulated in the aged skin. A reduced epidermal permeability barrier, including lower SC lipid levels, was also observed in the aged skin. Epidermal lipid amounts were increased in WT mice upon the INHI treatment, compared to the case for the mice in the vehicle group. Therefore, we could conclude that increased 11β-HSD1 expression in the aged skin could increase active-GC levels, which, in turn, reduce the SC lipid levels and lead to the deterioration of the epidermal permeability barrier. These results should encourage further research and development of topical or systemic drugs that inhibit 11β-HSD1 activity in the aging skin.

## 4. Materials and Methods

### 4.1. The Human Experiment

The clinical study protocol was approved by the Institutional Review Board of the Wonju Severance Christian Hospital (CR317026) and was performed in accordance with their guidelines. Informed consent was obtained from all participants. Ten healthy young (<25 years old; mean age, 21.6 years) and ten aged (>60 years old; mean age, 65.9 years) women were recruited for the study. The cortisol levels in their SC and oral epithelium were measured.

### 4.2. The Animal Experiment

The animal study protocol was approved by the Institutional Animal Care and Use Committee of the Yonsei University Wonju College of Medicine (YWC-150303-1) and was performed in accordance with the ARRIVE guidelines. Female hairless mice (*hr*/*hr*) were purchased from OrientBio (Seongnam, Republic of Korea). Experiments were conducted on five young (8-weeks-old) and five aged (56-week-old) female mice. Skin barrier function, serum cytokine levels, and 11β-HSD1 expression (via IHC) were measured. Additionally, 8- and 56-week-old *HSD11B1* KO mice and their WT counterparts (C57BL/6) were subjected to the following experiments. Either vehicle (dimethyl sulfoxide, DMSO) or INHI (38558, Merck, Readington Township, NJ, USA) dissolved in the vehicle was applied onto the backs of the mice twice a day for 10 days. The experimental groups were as follows: group 1, *HSD11B1* KO aged mice treated with vehicle (n = 11); group 2, WT aged mice treated with vehicle (n = 5); group 3, WT aged mice treated with INHI (n = 5); group 4, WT young mice treated with vehicle (n = 5); and group 5, WT young mice treated with INHI (n = 5). After 10 days of treatment, the SC corticosterone levels were measured, the CD density was determined by EM, and the epidermal lipid levels were analyzed.

### 4.3. Preparation of 11β-HSD1 KO Mice

Global *HSD11B1* KO mouse embryos were obtained from Professor Gareth G. Lavery (University of Birmingham, UK). Clones of the *HSD11B1* KO gene were injected into C57BL/6 blastocysts according to a previous report [64]. The embryos were transferred into pseudo-pregnant C57BL/6 female mice, and heterozygous mice were generated. To confirm the germline transmission of the KO allele, a conventional polymerase chain reaction was carried out. The following primers were employed in multiplex mode: P1, 5′-GGGAGCTTGCTTACAGCATC-3′; P2, 5′-CATTCTCAAGGTAGATTGAACTCTG-3′; and P3, 5′-TCCATGCAATCAACTTCTCG-3′. Primers P2 and P3 yielded a band of 139 bp, indicating the presence of the WT allele. Amplicons representing the amplification of the DNA fragment located between P1- and P3-binding sites were not detected owing to the distance between these primers. In the KO allele, a P2- binding site was removed to ensure proximity between the P1- and P3-binding sites, resulting in the generation of a 242 bp amplicon for the detection of the WT, heterozygote, and homozygote. Homozygous *HSD11B1* KO mice were obtained after several subsequent breeding steps.

### 4.4. Quantification of Cortisol and Corticosterone by ELISA

We collected human SC samples from the forearms of our participants by stripping off D-Squame disc tape (CuDerm, Dallas, TX, USA) from their skin. Mucosal epithelial samples were collected from the buccal mucosa using Isohelix buccal swabs (Cell Projects, Kent, UK). SC samples of dorsal skin were collected from the *HSD11B1* KO and WT mice by stripping ff D-Squame disc tapes from their skin before they were euthanized. The samples were placed in 500 µL of lysis buffer, vortexed, and incubated overnight at 4 °C. Cortisol and corticosterone levels in the obtained protein extracts were measured using the corresponding ELISA kits (Merck Millipore, Darmstadt, Germany) [45].

### 4.5. Measurement of Skin Barrier Function

Skin barrier function was assessed as the basal TEWL, SC hydration, and SC integrity (defined as changes in TEWL after 15 rounds of D-Squame disc tape stripping) of the dorsal skin of hairless mice. The TEWL was quantified with a Tewameter TM 210 (Courage and Khazaka Electronic GmbH, Cologne, Germany). SC hydration was measured using a Corneometer CM 850 (Courage and Khazaka) [65,66].

### 4.6. IHC Staining and Semi-Quantitative Analysis of 11β-HSD1

Dorsal skin samples from young and aged hairless mice were immunohistochemically stained for 11β-HSD1 [45]. Briefly, a primary antibody against 11β-HSD1 (Santa Cruz Biotechnology, Santa Cruz, CA, USA) was added to 4 μm-thick paraffin-embedded skin tissue sections, followed by incubation overnight at 4 °C. After three cycles of washing, the tissue sections were incubated with the appropriate secondary antibody for 30 min at room temperature (22–26 °C). Antigen–antibody complexes were visualized by staining with the ABC-HRP Kit (Vector Lab, Burlingame, CA, USA), and counterstaining was performed with hematoxylin [45,67]. This procedure was followed by a semi-quantitative analysis of staining intensity in the tissue sections stained for 11β-HSD1. The intensity was classified as follows: 4, marked; 3, moderate; 2, slight; and 1, basal.

### 4.7. Serum Cytokine Level Assays

In hairless mice, the serum levels of cytokines IL-1α, IL-4, IL-10, and IL-31, and TNF-α were measured using a bead-based multiplex immunoassay. Serum samples were centrifuged at 2000× *g* for 20 min to remove debris. Then, 75 μL of each serum sample and 75 μL of calibrator diluent RD6-52 were mixed at a 2-fold dilution. The serum levels of IL-1α, IL-4, IL-10, IL-31, and TNF-α were measured using the Magnetic Luminex Screening Assay Kit (R&D Systems, Minneapolis, MN, USA). The samples and the antibody cocktail were mixed and incubated for 2 h at room temperature (22–26 °C), followed by analysis on a Luminex 100 device (Luminex, Austin, TX, USA).

### 4.8. Quantitative EM

Skin biopsy samples were collected from *HSD11B1* KO mice and WT mice. The samples were pulverized into pieces less than 0.5 mm^3^, fixed overnight in modified Karnovsky’s fixative, and post-fixed in 2% aqueous osmium tetroxide containing 1.5% potassium ferrocyanide, similar to the protocols described in previous reports [11,68]. Next, the samples were dehydrated in ethanol and embedded in an Epon-epoxy mixture. Ultrathin skin sections were examined under a transmission electron microscope (JEM-1200EXII, JEOL, Tokyo, Japan) operated at 80 kV.

For the quantitative EM analysis of the CD, its densities in the EM images were analyzed via an objective method to exclude subjective bias. To evaluate CD density, which reflects SC integrity, we determined the CD length randomly from the first and second cell layers of the lower SC. The ratio of the total length of the CD to the total length of cornified envelopes in the lower SC was then calculated [11,69].

### 4.9. Lipid Analysis in the SC

Murine SC samples were collected by stripping off the D-Squame tape applied onto the back of the mice. SC samples were harvested from these D-Squame tapes and lysed in radioimmunoprecipitation assay buffer, and next, sphingolipids were extracted as per the procedures we have described previously [70,71]. The extracted lipids were dried in a vacuum system (Vision, Seoul, Republic of Korea), re-dissolved in methanol, and analyzed by liquid chromatography–electrospray ionization–tandem mass spectrometry (LC-ESI-MS/MS; API 3200 QTRAP mass; AB/Sciex, Framingham, MA, USA) in the selective ion monitoring mode. The ceramide MS/MS transitions (m/z) were 510→264 for C14-ceramide, 538→264 for C16-ceramide, 552→264 for C17-ceramide, 566→264 for C18-ceramide, 594→264 for C20-ceramide, 648→264 for C24:1-ceramide, 650→264 for C24-ceramide, 676→264 for C26:1-ceramide, and 678→264 for C26-ceramide.

To measure the free-cholesterol and free-FA levels, lipid extraction was performed using by the Folch method, with minor modifications [72,73,74]. Briefly, the SC tissues on the D-Squame tapes were lysed and sonicated in methanol–chloroform (1:2, *v*/*v*) containing butylated hydroxytoluene (500 µg/mL), followed by the addition of 500 pmol of docosahexaenoic acid and cholesterol-d_6_ as an internal standard. The extracted lipids were dried in the vacuum system, re-dissolved in methanol, and analyzed via LC-ESI-MS/MS, which was operated in the selective ion monitoring mode. First, free cholesterol and FAs were separated by reverse-phase high-performance liquid chromatography (a NANOSPACE SI-2 HPLC system equipped with an HTS autosampler Z, Shiseido, Tokyo, Japan) on a Kinetex C18 column (2.1 × 50 mm, internal diameter: 2.6 μm; Phenomenex, St. Louis, MO, USA), as described in previous studies [74,75]. The FA MS/MS transitions (*m*/*z*) were 227→183 for C14:0 FA, 253→209 for C16:1 FA, 255→211 for C16:0 FA, 277→233 for C18:3 FA, 279→235 for C18:2 FA, 281→237 C18:1 FA, 283→239 C18:0 FA, 303→259 for C20:4 FA, 311→267 for C20:0 FA, 337→293 for C22:1 FA, 339→295 for C22:0 FA, 365→321 for C24:1 FA, and 367→323 for C24 FA. The MS/MS transitions (*m*/*z*) were 369.3→161.5 for cholesterol, 369.3→147.1 for free cholesterol, and 374.4→152.7 for cholesterol-d_6_. All data were acquired using the Analyst 1.5.1 software (Applied Biosystems, Foster City, CA, USA).

### 4.10. Statistical Analysis

Either the Student’s *t*-test or the Mann–Whitney U test was performed, as appropriate, to analyze the differences between two groups using the GraphPad Prism 5 software (GraphPad Software, La Jolla, CA, USA). We performed two-way analysis of variance (ANOVA) followed by the Dunn–Bonferroni test to investigate the differences among multiple treatment groups. Statistical significance was set at *p* < 0.05.

## Data Availability

The datasets generated and/or analyzed during the current study are available from the corresponding author upon reasonable request.

## Supplementary Materials

The following are available online at https://www.mdpi.com/article/10.3390/ijms22115750/s1.
