# Supplementary material for: Increased Expression of 11β-Hydroxysteroid Dehydrogenase Type 1 Contributes to Epidermal Permeability Barrier Dysfunction in Aged Skin
Source: Int J Mol Sci. 2021 May 27;22(11):5750. doi: 10.3390/ijms22115750 (PMC8198579; doi:10.3390/ijms22115750)
Supplement: Supplementary file 1 [file ijms-22-05750-s001.zip › ijms-1218586-supplementary.pdf]

Table S1. *p*-values of the *post hoc* analysis presented in Figure 5a (stratum corneum corticosterone).

	Mean ± SD	KO aged veh	WT aged veh	WT aged INHI	WT young veh	WT young INHI
**KO aged veh**	3987.51 ± 922.88	N/A	0.069	0.320	0.827	>0.999
**WT aged veh**	5461.94 ± 1465.21		N/A	0.008	0.022	0.151
**WT aged INHI**	3373.46 ± 333.61			N/A	>0.999	0.548
**WT young veh**	4244.20 ± 1741.36				N/A	>0.999
**WT young INHI**	4211.6 ± 1297.88					N/A

SD, standard deviation; KO, knock-out; WT, wild-type; INHI, 11β-HSD1 inhibitor; veh, vehicle; N/A, not applicable.

Table S2. *p*-values of the *post hoc* analysis presented in Figure 5b (corneodesmosome density).

	Mean ± SD	KO aged veh	WT aged veh	WT aged INHI	WT young veh	WT young INHI
**KO aged veh**	2.39 ± 0.87	N/A	0.033	0.202	0.281	0.097
**WT aged veh**	1.00 ± 0.15		N/A	0.036	0.194	0.183
**WT aged INHI**	1.80 ± 0.72			N/A	0.833	0.755
**WT young veh**	1.84 ± 1.08				N/A	0.955
**WT young INHI**	1.63 ± 0.69					N/A

SD, standard deviation; KO, knock-out; WT, wild-type; INHI, 11β-HSD1 inhibitor; veh, vehicle; N/A, not applicable.

Table S3. *p*-values of the *post hoc* analysis presented in Figure 6a (ceramides).

	Mean ± SD	KO aged veh	WT aged veh	WT aged INHI	WT young veh	WT young INHI
**KO aged veh**	234.96 ± 42.03	N/A	0.530	0.343	0.003	0.003
**WT aged veh**	212.77 ± 27.75		N/A	0.008	0.008	0.008
**WT aged INHI**	259.56 ± 10.78			N/A	0.008	0.008
**WT young veh**	363.26 ± 42.52				N/A	0.056
**WT young INHI**	442.53 ± 52.31					N/A

SD, standard deviation; KO, knock-out; WT, wild-type; INHI, 11β-HSD1 inhibitor; veh, vehicle; N/A, not applicable.

Table S4. *p*-values of the *post hoc* analysis presented in Figure 6a (cholesterol).

	Mean ± SD	KO aged veh	WT aged veh	WT aged INHI	WT young veh	WT young INHI
**KO aged veh**	48.21 ± 4.83	N/A	0.639	0.048	0.005	0.003
**WT aged veh**	49.72 ± 3.20		N/A	0.095	0.016	0.008
**WT aged INHI**	56.71 ± 5.58			N/A	0.310	0.016
**WT young veh**	60.16 ± 5.50				N/A	0.056
**WT young INHI**	67.67 ± 4.88					N/A

SD, standard deviation; KO, knock-out; WT, wild-type; INHI, 11β-HSD1 inhibitor; veh, vehicle; N/A, not applicable.

Table S5. *p*-values of the *post hoc* analysis presented in Figure 6a (total fatty acids).

	Mean ± SD	KO aged veh	WT aged veh	WT aged INHI	WT young veh	WT young INHI
**KO aged veh**	48.21 ± 4.83	N/A	0.073	0.003	0.003	0.003
**WT aged veh**	49.72 ± 3.20		N/A	0.008	0.008	0.008
**WT aged INHI**	56.71 ± 5.58			N/A	>0.999	0.421
**WT young veh**	60.16 ± 5.50				N/A	0.310
**WT young INHI**	67.67 ± 4.88					N/A

SD, standard deviation; KO, knock-out; WT, wild-type; INHI, 11β-HSD1 inhibitor; veh, vehicle; N/A, not applicable.
